# A chaotic self-oscillating sunlight-driven polymer actuator

**DOI:** 10.1038/ncomms11975

**Published:** 2016-07-04

**Authors:** Kamlesh Kumar, Christopher Knie, David Bléger, Mark A. Peletier, Heiner Friedrich, Stefan Hecht, Dirk J. Broer, Michael G. Debije, Albertus P. H. J. Schenning

**Affiliations:** 1 Department of Chemical Engineering and Chemistry, Eindhoven University of Technology, PO Box 513, 5600 MB Eindhoven, The Netherlands; 2 Department of Chemistry and IRIS Adlershof, Humboldt-Universitat zu Berlin, Brook-Taylor-Strasse 2, 12489 Berlin, Germany; 3 Department of Mathematics and Computer Science, Eindhoven University of Technology, PO Box 513, 5600 MB Eindhoven, The Netherlands; 4 Institute for Complex Molecular Systems, Eindhoven University of Technology, PO Box 513, 5600 MB Eindhoven, The Netherlands

## Abstract

Nature provides much inspiration for the design of materials capable of motion upon exposure to external stimuli, and many examples of such active systems have been created in the laboratory. However, to achieve continuous motion driven by an unchanging, constant stimulus has proven extremely challenging. Here we describe a liquid crystalline polymer film doped with a visible light responsive fluorinated azobenzene capable of continuous chaotic oscillatory motion when exposed to ambient sunlight in air. The presence of simultaneous illumination by blue and green light is necessary for the oscillating behaviour to occur, suggesting that the dynamics of continuous forward and backward switching are causing the observed effect. Our work constitutes an important step towards the realization of autonomous, persistently self-propelling machines and self-cleaning surfaces powered by sunlight.

## Abstract (web summary)

It is highly desirable, yet challenging to build actuators in a dry environment that can undergo autonomous oscillation. Here, Kumar *et al.* achieve this goal in a soft actuator based on the use of a nematic liquid crystal film doped by ortho-fluoroazobenzene that is responsive to sunlight.

## Graphical abstract

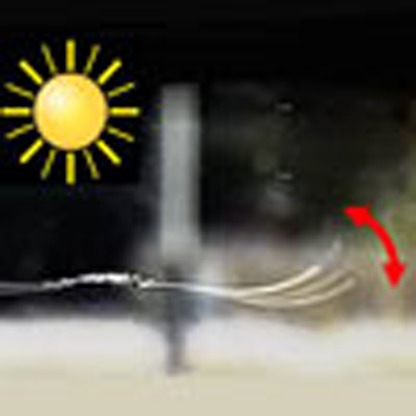

Autonomous oscillations are characteristic of living systems: the heartbeat, cell cycling and biorhythms are all oscillatory events. These continuous energy-driven actions do not require any switching on/off of external stimuli; rather, they are powered and regulated by chemical energy, and can sometimes be influenced by external physical cycles (circadian rhythms). A limited number of attempts have been made to create artificial systems mimicking this non-equilibrium behaviour. Almost all of them employ stimuli-responsive hydrogels in which periodic swelling and de-swelling based on water diffusion occurs. These systems are chemically driven either by pH oscillation or the Belousov–Zhabotinsky reaction^1^,^2^,^3^,^4^, and a variety of biomimetic actuators^5^, such as self-walking gels^6^ and photo-regulated wormlike motion^7^, have been reported. It remains a challenge to create actuators in a dry environment capable of autonomous oscillations, which are driven remotely by either artificial visible light or, ideally, the sun acting as the non-invasive power supply. If such autonomous systems can be realized using synthetic polymers, unprecedented, solar powered, continuously moving materials and devices should emerge, which are attractive for self-cleaning coatings and surfaces, able to actively prevent adhesion of particles and (bio)chemical in various outdoor applications, as well as solar powered soft robotics.

Light-driven soft actuators that operate in solvent-free environments have been studied in organic crystals^8^, inorganic crystals^9^, polymer brushes^10^ and liquid crystalline networks (LCNs)^11^,^12^,^13^,^14^,^15^. Most of them are based on azobenzenes acting as versatile photoreversible molecules that require ultraviolet and blue light to induce *trans*→*cis* and *cis*→*trans* isomerizations, respectively. For example, crawling crystals based on azobenzene have been shown to respond to the displacement of a continuous ultraviolet and visible light source because of periodic crystallization and melting at the front and rear edges of the crystal^16^. A LCN polymer belt containing azobenzenes has also been reported as a continuous light-driven motor powered by exposing the material to ultraviolet and visible light in two different areas^17^. Continuous oscillating cantilevers with high frequencies and large amplitudes formed from a planar aligned LCN film containing azobenzene derivatives fueled by a steady, high-intensity laser light source have also been described^18^,^19^. These latter polymer films were mounted vertically and first bent towards the specifically positioned laser source and subsequently started oscillating in plane to the long axis of the actuator surface as the front and back surfaces were alternately illuminated. Interestingly, sunlight—but only if concentrated by a lens system—could also drive the oscillation^19^.

In most azobenzene-based actuators, the use of high-energy ultraviolet light is required and thereby potentially limits the device lifetime by degrading the azobenzene and damaging its surroundings. It is thus highly desirable to tune the wavelength of photoisomerization to visible wavelengths^20^,^21^,^22^,^23^,^24^,^25^. Recently, fluorinated azobenzene molecules have been reported that can be switched solely by visible light, using wavelengths corresponding to blue and green colours, and that display remarkable stability in solution^26^,^27^,^28^. In this work we develop a visible light responsive soft actuator based on a LCN using a photopolymerizable *ortho*-fluoroazobenzene (F-azo) as the photoresponsive moiety incorporated into a nematic polymer network (Fig. 1). The film displays a unique, chaotic self-oscillation when exposed to ambient, non-concentrated sunlight (or a combination of common green and blue LEDs) and requires no variation in either the source or location of illumination to maintain constant motion.

## Results

### Producing the films

**Figure 1:**
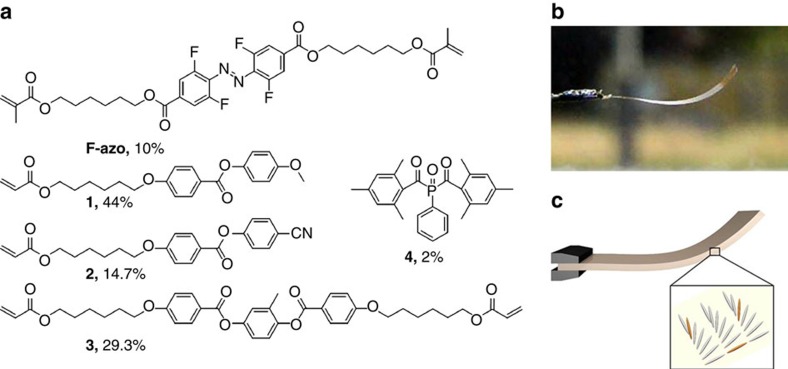
Components and structure of the F-azo polymer film. (**a**) Chemical structures of components used to prepare the nematic liquid crystalline network. (**b**) Photograph of splay-oriented film after removal from the cell under ambient interior light (homeotropic side on top) and (**c**) schematic of the LC splay aligned film (grey) containing F-azo molecules (orange).

The materials (monomers, crosslinker and initiator) used to produce the actuator films are shown in Fig. 1a. To obtain a bending actuator, splay-oriented films were utilized (Fig. 1c)^12^. Splay orientation allows increased bending of an actuator due to the non-uniform expansion/contraction effects at the two opposite sides of the film. The films were made using cells with one rubbed planar polyimide and one homeotropic polyimide-coated plate glued together using 18 μm spacers and filled by a LC mixture of the monoacrylates **1** and **2** and diacrylate **3** in a 3:1:2 weight ratio, 10 wt% of the F-azo dimethacrylate (synthesis details are included in the Methods section) with 2 wt% of photoinitiator **4**. The F-azo dye mixed well with the LC mixture and 10 wt% was used to have sufficient absorption in the visible region while minimizing the absorption gradient in the 18 μm thick layer. After filling, the cell was photopolymerized in the nematic phase (45 °C) for 30 min followed by a post heating treatment at 120 °C for 5 min to ensure complete polymerization (Supplementary Fig. 1).

### Characterizing the F-azo LC films

After opening the cell, the pristine 16 × 1.5 × 0.02 mm film bent in the direction of the homeotropic polymer side (Fig. 1b). This bending is probably the result of photopolymerization under ultraviolet light causing a certain degree of *cis* population, which once converted back to the *trans* isomer after the thermal treatment, leads to an increased order in the polymer film at room temperature and causes stress. This structural change leads to expansion at the planar side and shrinkage at the homeotropic top, resulting in the curl when the film was removed from the cell (Fig. 1c; Supplementary Fig. 2). The monodomain, splay orientation was confirmed by polarized attenuated total reflectance infrared spectroscopy (ATR-IR) where the stretching vibrations of the aromatic rings are used to determine the orientation of the LC network, observing the films under crossed polarizers, and via ultraviolet/Vis spectroscopy (Supplementary Figs 3 and 4)^29^. A glass transition temperature (*T*_g_) of ∼58 °C for the polymer film is determined from the peak maximum of tan delta curve obtained from dynamic mechanical thermal analysis (DMTA) (Supplementary Fig. 5). The order parameter, or *S* value, defined as


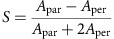


where *A*_par_ and *A*_per_ are the measured absorption of light polarized parallel and perpendicular to the LC alignment direction, respectively, are determined to be around *S*=0.23 at the bottom (more planarly aligned surface) and approximately *S*=0.03 at the top, more homeotropic surface, suggesting a splay film. The order parameter determined for the F-azo is low but most likely the result of the continuous isomerization during the photopolymerization step.

### Exposure to sunlight

**Figure 2:**
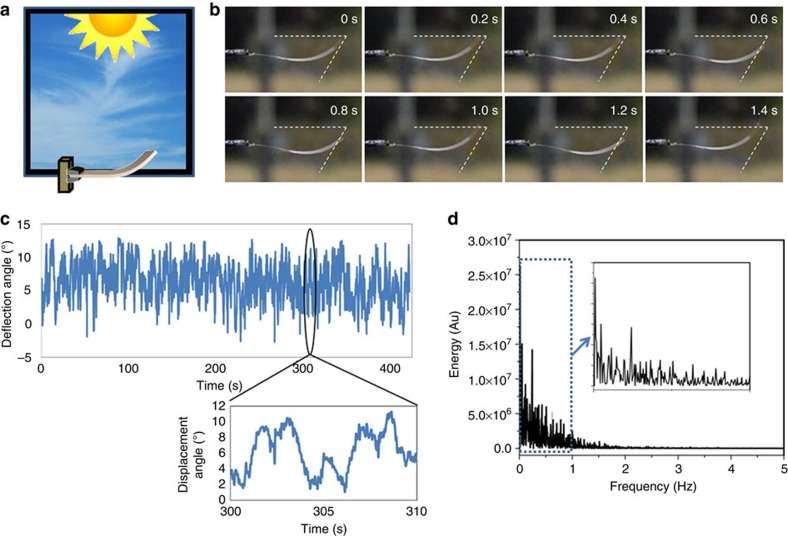
Continuous chaotic oscillations of the F-azo polymer film in sunlight. (**a**) Experimental setup for measurement of oscillatory motion of a photomechanical film during sun exposure. (**b**) Series of snapshots extracted from the video depicting film oscillations; dotted lines have been added to aid the eye. (**c**) Plot of the deflection angle versus time during sunlight exposure through the window derived from frame-by-frame analysis of the video: the inset is a blowup showing details from a 10 s period. (**d**) Frequency spectrum of the angle time series shown in 2c (inset: zoom of frequency spectrum between 0 and 1 Hz).

When the film is exposed to daylight it shows continual bending. To study this remarkable behaviour in more detail, the film was suspended and placed inside a south facing window on a sunny day (Fig. 2a). The film immediately begins to bend in an apparently chaotic oscillatory fashion: the film wavers up and down with no easily observable regular frequency, and when held vertically, it flexes from left to right in a similar fashion. An increase in temperature from 24 °C (ambient) to 31 °C under sunlight exposure is measured by an infrared camera. The total solar light intensity on the sample is estimated using Spectral2 from NREL and window transmission measurements to be about 35 mW cm^−2^. To analyse this behaviour in more detail, the bending action was recorded (see Supplementary Video 1, showing the first 30 s of a 7 min movie) and a series of snapshots depicting the action is shown as Fig. 2b. Each frame of the movie spaced 0.033 s apart was analysed with respect to the angle of deflection, given by the suspension point and the tip end of the film and the displacement distance of the film tip (Supplementary Fig. 6). The corresponding data of the entire movie is plotted in Fig. 2c. The polymer film showed a fully reversible and continuous actuation with bending angles ranging between −2 and 12°. Time series analysis of the deflection angle data by discrete Fourier transformation^30^ gives a frequency spectrum (Fig. 2d) that reveals that the fluctuation is a combination of a small-amplitude white-noise component superimposed on a signal that fills the frequencies up to about 1 Hz (Supplementary Fig. 7). This suggests that the angle fluctuations are generated by some process, which is probably nonlinear and definitely appears to be chaotic.

### Comparing F-azo and H-azo

To rule out external air currents or other artefacts causing the oscillations, a film containing a standard, non-fluorinated azobenzene in which the fluorine atoms had been replaced by hydrogen (H-azo, Fig. 6 in the Methods section)^12^ with an identical LC mix with splay alignment is prepared and mounted beside the F-azo film. The response of these two adjacent films to sunlight was recorded (Supplementary Video 2). Again, the F-azo film oscillates continuously with the same characteristics as shown before whereas the H-azo film does not move at all in the sunlight. This indicates the bending behaviour originates from the photoresponsive F-azo units in the polymer film, and is not the result of external air currents or other artefacts.

### Exposure to a solar simulator

**Figure 3:**
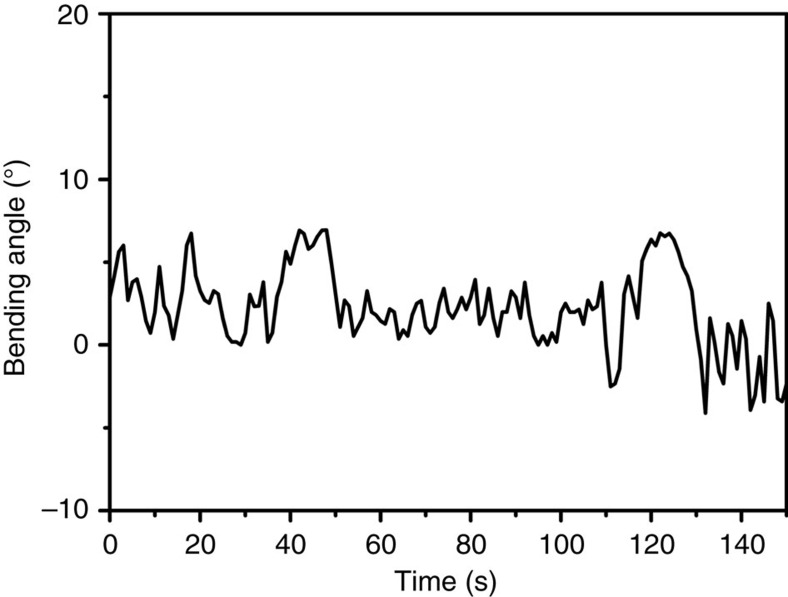
Solar simulator exposure of the F-azo polymer films. Plot of the measured deflection angle as a function of time, derived from frame-by-frame analysis of the video (every 30th frame analysed corresponding to a 1 s increment).

While it is unlikely that exterior atmospheric conditions could cause sunlight intensity or spectral range and distribution to constantly change on timescales in the range of seconds, to rule out this possibility the samples were studied in a controlled environment. Samples suspended homeotropic side up and illuminated by a 300 W solar simulator equipped with 1.5 AM (global) solar spectrum (LOT-Oriel) filters emitting 5.57 mW cm^−2^ from 485 to 560 nm and 2.02 mW cm^−2^ from 385 to 435 nm from a distance of about 10 cm (Fig. 3a) immediately exhibit self-oscillating behaviour under these constant lighting conditions. The oscillatory motions were recorded with a video camera. While the amplitude of the oscillations seemed regulated by the light intensity (the power provided to the lamp was varied from 210 to 295 W), we detect no significant changes in the oscillation frequencies of the films. The bending response is plotted as a function of time (Fig. 3b), revealing that the F-azo film produced a nonlinear oscillating response similar to the sun as stimulus. This affirms that the continuous self-oscillation takes place under constant sunlight conditions.

### Exposure to blue and green LED lights

**Figure 4:**
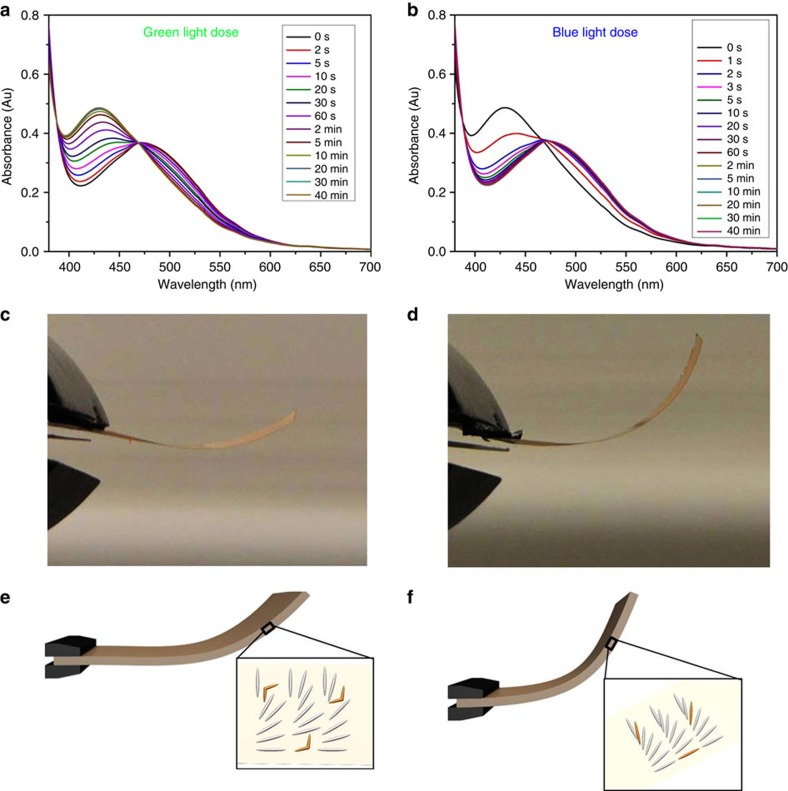
Visible light exposure of the F-azo polymer films. Ultraviolet–vis absorption spectra of the planar aligned polymer film as a function of illumination time when exposed to light of (**a**) 530 nm and (**b**) 405 nm wavelength. The UV absorption bands at 430 and 470 nm correspond to the *n*→π* transitions of *cis* and *trans* isomers, respectively. Photographs of the splay-oriented film after illumination by light of (**c**) 530 nm and (**d**) 405 nm wavelengths. Below the photographs in **e** and **f** are representations of the molecular order of the films showing the mesogen (grey) and the F-azo molecules (orange).

To gain more insight into the origin of the sun-driven actuation, the photoisomerization of the F-azo in the LC networks was monitored during exposure to monochromatic blue (405 nm) and green light (530 nm) from LEDs from Thorlabs using a planar aligned polymer film (Fig. 4a,b). After production, the polymer film kept in the dark shows an absorption maximum at 470 nm, corresponding to the *n*→π* transition of *trans* F-azo isomer^27^,^28^. When the film is exposed to green light the absorption maximum at 470 nm decreases while a new absorption band at 430 nm appears, corresponding to the *n*→π* transition of the *cis* isomer. After 2 min exposure a photostationary state is reached and the absorption band at 470 nm has disappeared. As the separation of the n–π* bands of *trans* and *cis* isomers in solution is 50 nm (ref. 27), such a 40 nm blue-shift indicates that a large fraction of the *trans* form is converted to the corresponding *cis* form. When exposed to blue light of 405 nm, the photo-induced *cis→trans* isomerization is much faster, resulting in complete photo-conversion in <10 s.

The influence of blue and green light on the bending behaviour of the splay actuator was also analysed. After exposure to 530 nm green light for a few seconds, the freestanding polymer film is almost flat (Fig. 4c). The generation of *cis* isomers results in a small decrease of the local molecular order, that is, the order parameter, leading to expansion at the homeotropic side and shrinkage at the planer side of the polymer film^12^. After exposure to 405 nm blue light, the bent state is attained again (Fig. 4d), showing that the process is fully reversible. It should be noted that during illumination by blue light the straight film first bends downwards partially due to reduction of the modulus of the film (Supplementary Fig. 2, *vide infra*). Our results indicate that the changes in molecular shape of the photoresponsive molecule in splay LCNs generate the reversible photo-induced bending in the bulk film and that the population of the *cis* and *trans* isomers determines the degree of bending. It should be noted that no discernable oscillation is observed during either of these illumination conditions (*vide infra*).

### Dual blue and green LED light exposure

**Figure 5:**
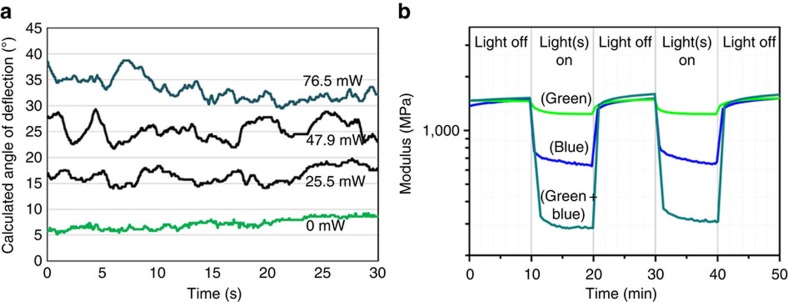
Requirement of simultaneous blue and green light for oscillation. (**a**) Plot of the calculated deflection angle versus time of F-azo film during exposure to 157 mW cm^−2^ green (530 nm) LED light and simultaneously to blue (405 nm) light of the intensity depicted in the graph, derived from frame-by-frame video analysis. (**b**) Storage modulus of the polymer film as a function of time during illumination to 157 mW cm^−2^ green (green line), 318.8 mW cm^−2^ blue (blue line), and both blue and green LEDs simultaneously (dark cyan line).

Finally, the films were placed equidistant from the two LED light sources, one emitting at 405 nm, the other at 530 nm, to investigate if a continually chaotically oscillating bending behaviour could also be obtained by dual visible light exposure. The intensity ratio incident on the film from the two lamps was gradually changed and the oscillation behaviour was recorded. For pure 405 nm blue or pure 530 nm green light illumination, there is little, if any, discernable oscillation after the initial bending (*vide supra*); it was noted the film tends to sag and bend downwards under illumination of the 405 nm LED. However, constant illumination employing different blue/green light intensity ratios results in increased oscillations. To substantiate and quantify the visual assessment, all individual frames of the 30 movies were analysed in terms of the displacement of the film tip as detailed in Supplementary Fig. 6 (a selection of this movie can be found as Supplementary Video 3), and the distance this tip travelled between movie frames in pixels was calculated. An example of the calculated angular displacement of the film tip for four different illumination conditions, one using only green LED light at 530 nm, and subsequent exposure to both green and 405 nm blue LED light at increasing intensities simultaneously, are displayed in Fig. 5a: absolute, cumulative displacement *vs* time for each light condition is found as Supplementary Fig. 8. This data strongly suggests that the oscillatory motion is caused by molecular isomerization reactions, as a proper mix of simultaneous green and blue light irradiation is necessary to generate the oscillatory behaviour.

Simple isomerization cannot by itself account for the continual oscillations: something else must be playing a role to achieve the chaotic behaviour, otherwise we would achieve a simple bending as has been described to date in other work^13^,^14^. To investigate which other factors play a role in the chaotic bending behaviour, the effect of illumination on the temperature and modulus of the polymer film were investigated. The storage modulus of the polymer networks was measured using DMTA during both green and blue LED light illumination and the temperature of the film was recorded with an infrared camera (Supplementary Fig. 9). When irradiated with green light, *trans*→*cis* isomerization of the F-azo units results in a small storage modulus decrease of the films from 1,450 to 1,250 MPa (Fig. 5b)^31^. After extinguishing the light source, the modulus quickly reverts to the initial value. When the film is illuminated with only blue light, the modulus decreased to 700 MPa, partly due to the *cis–trans* isomerization releasing stored energy as heat^32^,^33^. The modulus drastically reduced to 300 MPa when the film was exposed to combined blue and green light indicating enormous softening of the polymer. The softening of the film could be the explanation for the sag seen in the films under 405 nm light exposure. The film immediately reverts to its initial form after removal of the blue light source. In the latter illumination case the apparent surface temperature in the polymer film rises to 44 °C. The temperature increase cannot explain the decreases observed as the modulus at this temperature is still above 1 GPa (Supplementary Fig. 5). It suggests that the dual light-induced oscillating *trans↔cis* isomerizations of the azobenzenes and the corresponding molecular motion is causing the softening of the polymer film^27^,^28^,^34^,^35^. After switching off the light sources, the modulus rapidly returns to its initial state while the *cis* population is still present (Supplementary Fig. 10). This behaviour reveals that it is not the *cis–trans* population (thermodynamics) but rather the isomerization kinetics, inherent in the dynamic nature of the photostationary state, which is the dominant factor in this photosoftening process.

## Discussion

Besides the necessity of blue and green wavelengths, it is rather difficult to deduce further information about the requirements to achieve significant degrees of oscillation. Although it seems likely that the observed self-oscillating bending is caused at least partially by an oscillating population of *cis* and *trans* isomers, we are not able to clearly prove this as they are likely the result of minor population changes too small to be accurately measured via ultraviolet/Vis spectroscopy (Supplementary Fig. 10). As observed for the dual light exposure, sun induced softening of the polymer probably also takes place but to a lesser extent. This softening combined with local temperature changes is probably not homogenously distributed and varying in time throughout the film, which could cause fluctuations in local *cis–trans* populations and kinetics in the film. While the exact source of this erratic oscillatory motion is not yet completely understood, we postulate that the oscillations result from a coupling of periodic small changes of the *cis*–*trans* population and their isomerization rates, influenced by local heating and cooling^19^,^36^ as well as local photosoftening of the sample^27^,^28^,^34^,^35^,^37^. This shows that, similarly to self-oscillating gels^4^, a quantitative understanding of this irregular self-oscillation behaviour is quite challenging.

**Figure 6:**
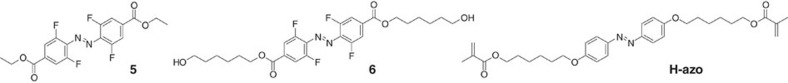
Structures of H-azo and precursors of F-azo.

As a preliminary study, a film containing a more standard, non-fluorinated azobenzene in which the fluorine atoms is replaced by hydrogen (H-azo, Fig. 6)^12^ with an identical LC mix with splay alignment was prepared and exposed simultaneously to a 29 mW cm^−2^ light from a 365 nm LED and 455 nm LED light at 35 mW cm^−2^ from a distance of 20 cm. Such conditions initiate continual, oscillatory motion, showing the universal nature of the dual exposure. A film depicting these experiments may be found as Video 4.

In conclusion, a self-oscillating sun and dual visible light responsive soft actuator is developed using *ortho*-fluoroazobenzenes as photoisomerizable molecules within a nematic liquid crystal polymer network. Normal actuation followed *trans*→*cis* and *cis*→*trans* isomerization of the photoswitch initiated by green and blue light, respectively. The film showed an unprecedented continuous chaotic oscillating motion upon exposure to non-concentrated sunlight and was reproduced by exposure to the combined light of blue and green LEDs. The continuous chaotic oscillation using two light stimuli simultaneously appears to be a general phenomenon since a very similar behaviour was observed upon dual ultraviolet/blue light irradiation using a regular (non-fluorinated) azobenzene derivative in the same liquid crystalline matrix (Supplementary Video 4) and should therefore be of great interest for fabricating self-oscillating soft actuators that continually function under constant light conditions. The motion is programmed by the molecular organization in the polymer film and is independent of the position of the light source. Interestingly, while our material mimics biological systems responding to sunlight, it is unique in that it uses sunlight as a constant energy source rather than a stimulus. Based on the observed unique behaviour we foresee immediate practical outdoor applications including self-cleaning coatings and surfaces and furthermore envision the future development of self-propelling/morphing soft actuators, operating in a dry environment and able to harvest and convert solar energy.

## Methods

### General synthetic and analytical methods

The commercial starting materials were used as supplied. The solvents were either used as received or dried employing an Innovative Technologies solvent purification system. Silica gel (Merck 60, particle size 0.040–0.063 mm) was used for column chromatography. NMR spectra were recorded on a Bruker 300 MHz (75 MHz for ^13^C, 282 MHz for ^19^F) spectrometer using residual protonated solvent signals as internal standards for ^1^H- and ^13^C-spectra (^1^H-NMR: *δ* (CDCl_3_)=7.26 p.p.m., *δ* (CD_2_Cl_2_)=5.32 p.p.m. and ^13^C-NMR: *δ* (CDCl_3_)=77.16 p.p.m., *δ* (CD_2_Cl_2_)=53.84 p.p.m.) or CFCl_3_ as external standards for ^19^F-spectra. Multiplicities are abbreviated as follows: singlet (s), doublet (d), triplet (t), quadruplet (q), quintet (quint), multiplet (m), and broad (br). Ultraperformance liquid chromatography coupled to mass spectrometry detection (UPLC-MS) was performed with a Waters Alliance system (gradient mixtures of acetonitrile/water) equipped with Acquity UPLC columns. The Waters system consisted of a Waters Separations Module 2695, a Waters Diode Array detector 996, a LCT Premier XE mass spectrometer, and a Waters Mass Detector ZQ 2000.

### Monomer synthesis and characterization

*E*-F4-diethylester (**5**). The synthesis of this compound is described in the literature^27^. ^1^H-NMR (300 MHz, CDCl_3_): *δ*=7.80–7.69 (m, 4H), 4.43 (q, J=7.1 Hz, 4H), 1.43 (t, *J*=7.1 Hz, 6H). ^13^C-NMR (75 MHz, CDCl_3_): *δ*=163.9–163.8 (m), 155.2 (dd, *J*=262.6, 3.9 Hz), 134.5–134.1 (m), 133.9 (t, *J*=9.3 Hz), 114.1 (dd, *J*=22.5, 3.5 Hz), 62.3 (s), 14.3 (s). ^19^F-NMR (282 MHz, CDCl_3_): *δ*=−118.88 (d, *J*=9.0 Hz). HRMS-ESI: *m/z*=399.0981 (calcd. for [M+H]^+^, 399.0968). *E*-F4-dihexanol (**6**). A mixture of **5** (0.81 g, 2.03 mmol, 1 eq), 1,6-hexanediol (4.80 g, 40.60 mmol, 20 eq), and K_2_CO_3_ (0.03 g, 0.20 mmol, 0.1 eq) in DMSO (14 ml) was placed in a flask on a rotary evaporator and subjected to 60 °C/50 mbar for 9 h, resulting in a continuous removal of MeOH formed during the transesterification reaction. Water and ethyl acetate were added and the layers were separated. The organic layer was washed with water (3 ×) and brine, dried over MgSO_4_, filtered, and concentrated under reduced pressure. The crude product was purified by flash column chromatography yielding azobenzene **6** as a red solid (0.75 g, 68%). ^1^H-NMR (300 MHz, CD_2_Cl_2_): *δ*=7.75 (d, *J*=9.3 Hz, 4H), 4.36 (t, *J*=6.6 Hz, 4H), 3.61 (t, *J*=6.4 Hz, 4H), 1.93–1.67 (m, 4H), 1.67–1.31 (m, 12H). ^19^F-NMR (282 MHz, CD_2_Cl_2_): *δ*=-119.73 (d, *J*=9.7 Hz). HRMS-ESI: *m/z*=543.2137 (calcd. for [M+H]^+^, 543.2118). ***E*-F4-azo**. Methacrylic acid (0.56 g, 6.65 mmol, 5 eq) and a catalytic amount of DMF were dissolved in dry DCM (5 ml) under argon. After cooling down to 0 °C, oxalyl chloride (0.51 ml, 5.98 mmol, 4.5 eq) was added and stirring was continued at room temperature until the evolution of gas stopped (ca. 3 h). The obtained methacryloyl chloride solution was slowly added to a mixture of **2** (0.72 g, 1.33 mmol, 1 eq), triethylamine (1.84 ml, 13.30 mmol, 10 eq), and DMAP (3 mg, 0.03 mmol, 0.02 eq) in dry DCM (5 ml). The resulting solution was stirred overnight, washed with water, saturated aqueous NaHCO_3_-solution (2 ×), and brine, dried over MgSO_4_, filtered and concentrated under reduced pressure. The crude product was purified by flash column chromatography yielding the azobenzene monomer (**F4-azo**) as a red solid (0.72 g, 80%). The compound was further recrystallized from a cyclohexane/chloroform mixture to obtain the crosslinker in high purity. ^1^H-NMR (300 MHz, CDCl_3_): *δ*=7.73 (m, 4H), 6.09 (m, 2H), 5.55 (m, 2H), 4.37 (t, *J*=6.6 Hz, 4H), 4.16 (t, *J*=6.6 Hz, 4H), 1.94 (m, 6H), 1.76 (m, 8H), 1.49 (m, 6H). ^13^C-NMR (75 MHz, CDCl_3_): *δ*=167.6, 163.9 (t, *J*=2.8 Hz), 155.2 (dd, *J*=262.8, 3.8 Hz), 136.6, 134.3 (t, *J*=10.2 Hz), 133.8 (t, *J*=9.5 Hz), 125.4, 114.1 (dd, *J*=22.5, 3.4 Hz), 66.2, 64.66, 28.65, 25.85, 25.80, 18.47. ^19^F-NMR (282 MHz, CDCl_3_): *δ*=−118.78 (d, *J*=9.1 Hz). HRMS-ESI: *m/z*=701.2469 (calcd. for [M+Na]^+^, 701.2462).

### Film production

Polymer films were fabricated using a mixture containing 44 wt% **1**, 14.7 wt% **2** and 29.3 wt% monomer **3**, 10 wt% **F-Azo**, 2 wt% photoinitiator **4**. Monomers **1** to **3** were obtained from Merck UK while photoinitiator **4** (Ciba Irgacure 819) was obtained from Ciba. **H-Azo** was custom-synthesized by Synthon (Germany). The monomers were mixed by dissolving in dichloromethane and dried at 50 °C (Fig. 6).

Glass substrates were cleaned by a 5 min dip in acetone and subsequently 2-propanol while stirring, flushed with demi water followed by drying with a nitrogen flow. To make a splayed molecular orientation one glass slide was coated with Polyimide A1051 (Sunever, Nissan Chemical, Japan) and baked at 180 °C followed by uniaxial rubbing. The other glass slide was coated with SE1211 polyimide for homeotropic alignment (Nissan) and baked at 200 °C. These two glass slides were glued together forming a cell with an 18 μm gap. The polymer film was prepared by filling the cell with the monomer mixture in the isotropic phase (80 °C) and slowly cooled down to nematic phase (35 °C). The photopolymerization was carried out at 35 °C for 30 min, followed by heating to 120 °C for 5 min to fully polymerize the film (Supplementary Fig. 1).

### Film characterization

The films were studied for liquid crystal alignment quality by observing light transmission through crossed polarizers using a Leica polarized optical microscope equipped with a Linkam hot stage. The alignment of both sides of the film was determined by polarized ATR-IR spectra. The films were marked appropriately, and in all the experiments described in the main text the film orientation was verified and recorded.

Polarized and normal absorption spectra were recorded on a Shimadzu UV-3102 Spectrometer Representative images of absorption spectra taken at various times in the film production are shown in Supplementary Fig. 11. The storage modulus and tan delta of the films were determined using a Q800 machine by TA Instruments. The surface temperatures of the F-azo films were recorded by a Thermacam T400 thermal camera (FLIR Systems) during illumination on the DMTA device.

Each frame of the recorded movies (full HD, NTSC, recorded on a Nikon D3200 SLR camera) was quantified with respect to the film tip position by image analysis. To this end, the movies recorded under different light conditions were imported into Matlab and each frame analysed.

### Data availability

The data that support the findings of this study are available from the corresponding author upon request.

## Additional information

**How to cite this article:** Kumar, K. *et al.* A chaotic self-oscillating sunlight-driven polymer actuator. *Nat. Commun.* 7:11975 doi: 10.1038/ncomms11975 (2016).

## Supplementary Material

Supplementary Information Supplementary Figures 1-11, Supplementary Table 1 and Supplementary Reference

Supplementary Movie 1 First 30 s of a 7 minute movie depicting the chaotic, oscillatory motion of an F-azo film exposed to regular sunlight on a window ledge within the Chemistry building on the Eindhoven University of Technology campus.

Supplementary Movie 2 30 s movie depicting the chaotic, oscillatory motion of an F-azo film exposed to regular sunlight mounted adjacent to an H-azo film placed on a window ledge within the Chemistry building on the Eindhoven University of Technology campus.

Supplementary Movie 3 Roughly 5 min of a longer movie depicting the effect of various intensities of light from a 405 nm blue and a 530 nm green LEDs on the oscillatory behavior of an F-azo film. The selected video displays the use of 155 mW/cm^2^ 405 nm and a variable intensity of 530 nm light. Subtitles in the movie detail the illumination intensity of the two LEDs during the experiment.

Supplementary Movie 4 16 s movie clip depicting the chaotic oscillatory behavior of an H-azo film exposed to 29 mW/cm^2^ light from a 365 nm LED and 35 mW/cm^2^ of 455 nm LED light.
