# Supplementary material for: A chaotic self-oscillating sunlight-driven polymer actuator
Source: Nat Commun. 2016 Jul 4;7:11975. doi: 10.1038/ncomms11975 (PMC4932179; doi:10.1038/ncomms11975)
Supplement: Supplementary Information — Supplementary Figures 1-11, Supplementary Table 1 and Supplementary Reference [file ncomms11975-s1.pdf]

Supplementary Figures

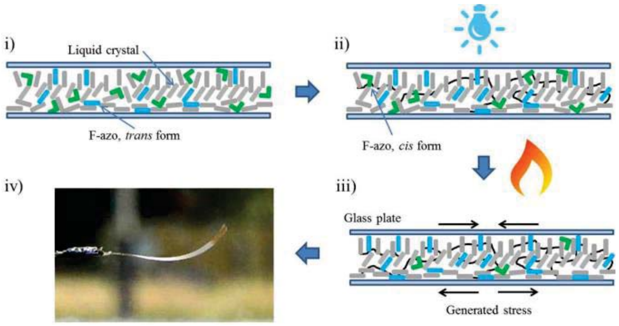

Supplementary Figure 1. Schematic setup for preparation of a splay aligned film. (i) Monomers used to fill the cell and (ii) exposed to UV light, initiating polymerization, followed by (iii) heating the sample to increase monomer conversion and simultaneously generating more trans fluorinated azobenzenes resulting in the generation of stresses within the film. (iv) Movie frame of a splay oriented film (homeotropic side on top).

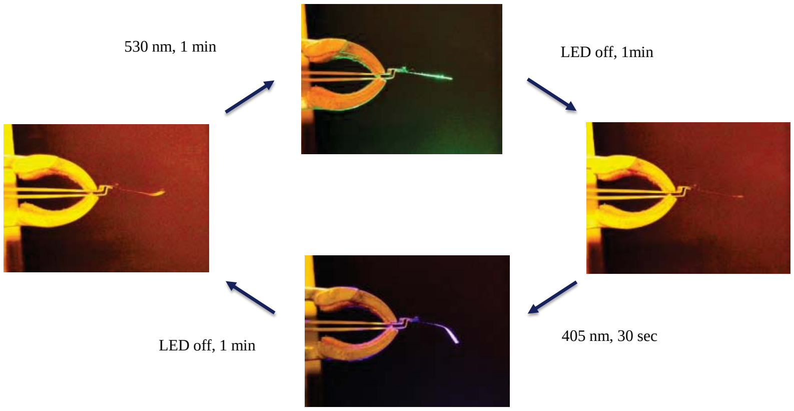

Supplementary Figure 2. Sequence of photographs of a typical F-azo sample under various lighting conditions in the two LED experiment. In the 'rest' state (left), that is, after exposure to UV light during the initial photopolymerization process, the film has a curl. Upon exposure to green light (center, top), the sample straightens and retains this shape after the light is extinguished (right). During exposure to blue 405 nm LED light (bottom, center), the sample tends to sag, and bend downwards. Immediately after removal of the light source, the film returns to its curled state.

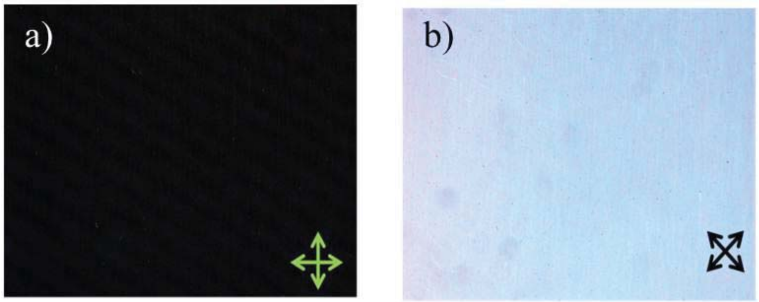

Supplementary Figure 3. Polarized optical microscopy photographs of the polymer network observed between crossed polarizers, with the arrows showing the polarization direction of the light: (a) director parallel to axis of one polarizer, and (b) the director at an angle of 45º to the axes of the polarizers.

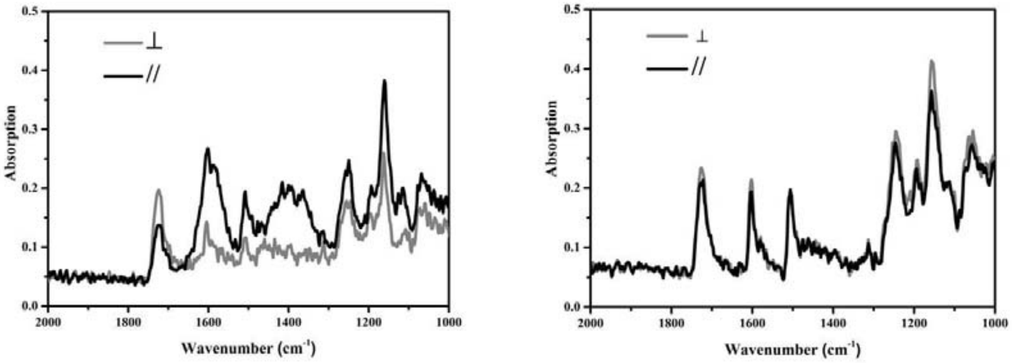

Supplementary Figure 4. Polarized attenuated total reflectance IR spectra of F-azo film at (left) planar surface side and (right) homeotropic side.

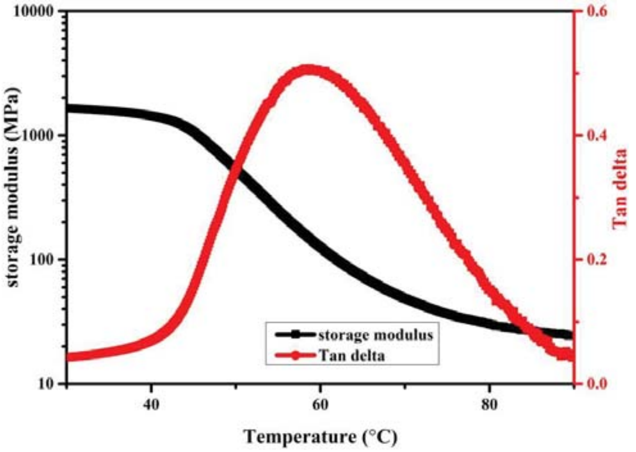

Supplementary Figure 5. Storage modulus and tan delta of the polymer film as a function of temperature as determined by DMTA.

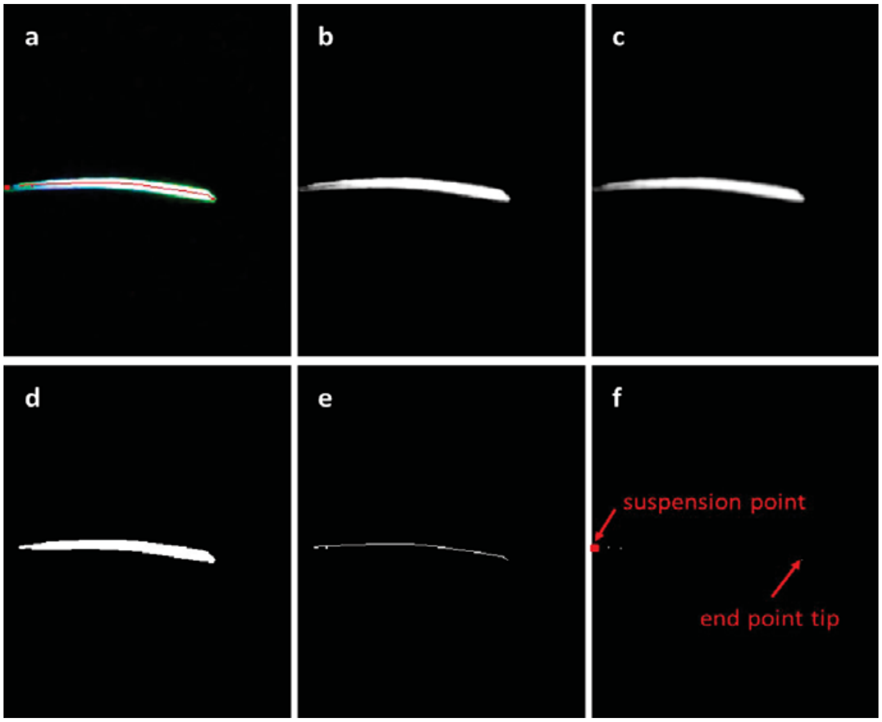

Supplementary Figure 6. Image analysis scheme applied to each frame of the movies (a) cropped region of interest in frame = raw color image; (b) grayscale image produced from color image preserving luminescence; (c) Gaussian filtered image with HWHM of 2 pixels; (d) segmentation of the filtered image using 1.3 times the Otsu threshold [1]; (e) skeletonization of the binary image; (f) end points of skeleton structure and manually selected static suspension point.

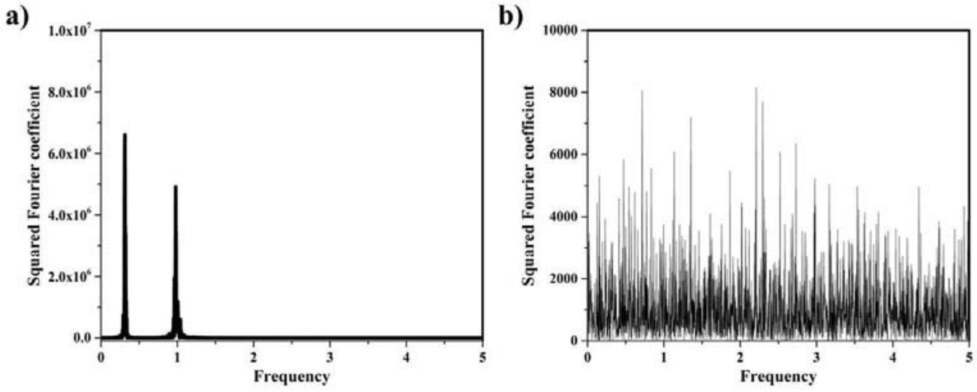

Supplementary Figure 7. Frequency spectra of two artificially generated time series for comparison to the angle time series obtained in Figure 2d of the main text. (a) A quasi-periodic (two-sine-wave) time series; (b) white noise. Both are constructed on the same set of time points as the original angle data found in the main text.

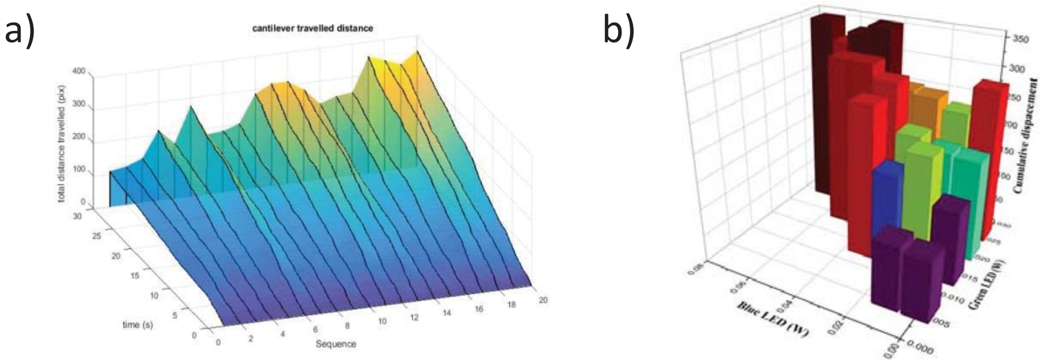

Supplementary Figure 8. (a) Cumulative tip deflection *vs* time *vs* different light conditions sequence over 30 frames of 1 second intervals (Details of the light conditions vs sequence number can be found in Supplementary Table 1 below). (b) Plot of absolute, cumulative displacement in pixels of the film tip over a period of 30 s as a function of actual 405 nm blue and 530 nm green incident LED power on the 16 x 1.5 mm^2 films.

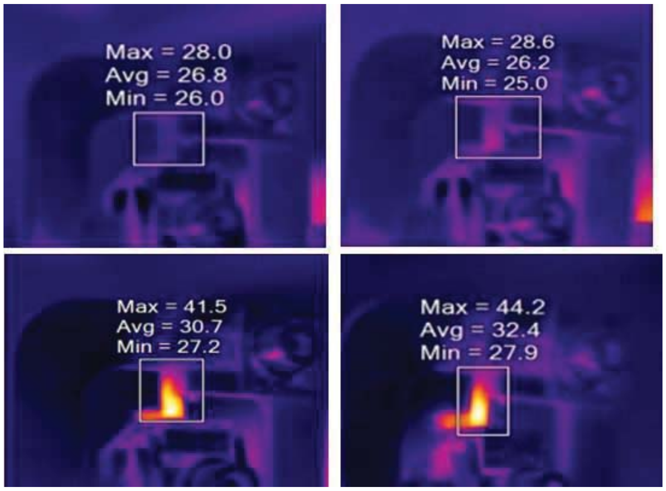

Supplementary Figure 9. IR images of the F-azo film in response to (a) room light, (b) 530 nm LED light, (c) 405 nm LED light, (d) and both 530 nm and 405 nm LED lights on the DMTA device.

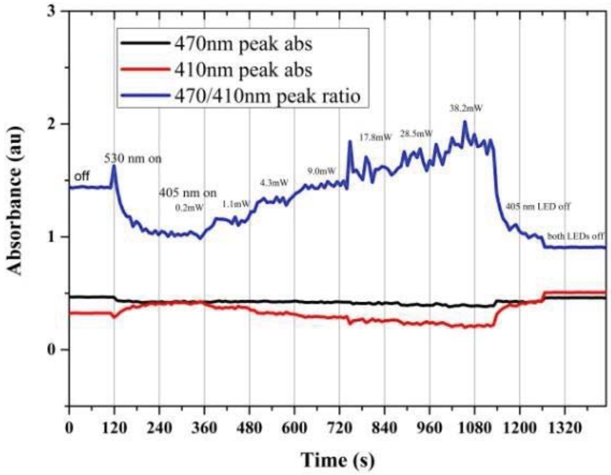

Supplementary Figure 10. Absorbance of the 470 nm peak (black line), 410 nm peak (red line), and ratio of the 470 nm to 410 nm peaks (blue line) plotted as a function of exposure time for increasing intensities of blue LED light at 405 nm under continual 530 nm green illumination.

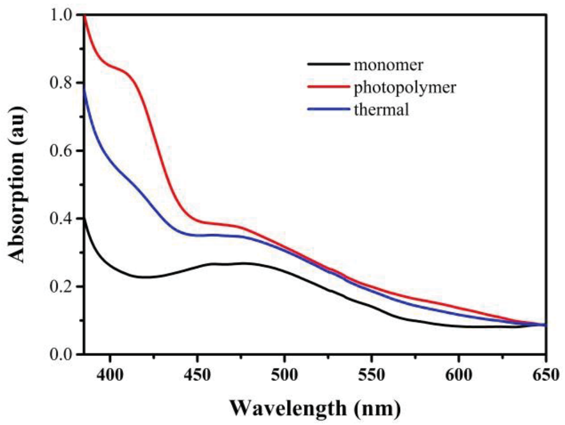

Supplementary Figure 11. The UV/Vis absorption spectra of the samples recorded at various stages of production. Absorbance spectra of the F-azo films before (black) and after (red) photopolymerization by exposure to UV light, and the spectrum of the film after the final thermal treatment (blue) displaying alterations in the relative contribution from the *cis*- and *trans*- states (see Figure 4 a,b in main text).

Supplementary Table

Supplementary Table 1. Data sequence for films exposed to blue and green LED light.

Sequence number	Video start time (s)	Video end time (s)	Green LED power (mW/cm^2)	Blue LED power (mW/cm^2)
1	00:25	00:55	24.16	0
2	00:59	01:29	24.16	55.4
3	01:43	02:13	58.3	0
4	02:16	02:46	58.3	55.4
5	02:54	03:24	58.3	106.3
6	03:31	04:01	58.3	152.9
7	04:12	04:42	85.4	0
8	04:47	05:17	85.4	55.4
9	05:25	05:55	85.4	106.3
10	06:00	06:30	85.4	152.9
11	06:34	07:04	85.4	199.6
12	07:10	07:40	85.4	240.8
13	07:56	08:26	107.9	0
14	08:33	09:03	107.9	55.4
15	09:10	09:40	107.9	106.3
16	09:45	10:15	107.9	152.9
17	10:21	10:51	107.9	199.6
18	10:56	11:26	107.9	240.8
19	11:32	12:02	107.9	280.4
20	12:07	12:37	107.9	318.8

Supplementary Reference

1 Otsu, N. A Threshold Selection Method from Gray-Level Histograms. *Systems, Man and Cybernetics, IEEE Trans.* **9**, 62-66 (1979).
